# Mechanism of gut microbiota and Axl/SOCS3 in experimental autoimmune encephalomyelitis

**DOI:** 10.1042/BSR20190228

**Published:** 2019-07-02

**Authors:** Xiao-Ling Li, Bo Zhang, Meng-Jiao Sun, Cai-Cai Bao, Bo-Yao Yuan, Qin-Fang Xie, Li-Juan Wang, Man-Xia Wang

**Affiliations:** 1 Department of Neurology, The Second Hospital of Lanzhou University, Lanzhou 730030, China; 2 Department of Cardiovascular Medicine, The First Hospital of Lanzhou University, Lanzhou 730000, China

**Keywords:** experimental autoimmune encephalomyelitis, Gut microbiota, Inflammatory inhibitors, Receptor tyrosine kinase subfamily

## Abstract

Multiple sclerosis (MS) is an immune-mediated disease of the central nervous system (CNS). The present study explored the role of intestinal microbiota in the initiation and propagation of mice induced by experimental autoimmune encephalomyelitis (EAE), an animal model of multiple sclerosis. 48 C57BL/6 were randomly divided into control group and EAE group. The changes of body weight and the scores of neurological function were recorded. The mRNA expression of the receptor tyrosine kinase subfamily (AXL) was detected by real-time quantitative PCR. The levels of IL-17 and IFN-γ in blood samples were examined by ELISA. The intestinal microbial composition of mice at different time points during the EAE induction was analyzed by 16S rRNA gene-based sequencing. In EAE group, the body weight began to reduce at day 3 and neurological symptoms began to appear at day 7 after EAE induction. The levels of IL-17 and IFN-γ in EAE group reached the peak at day 21 and then decreased gradually. However, the expression of Axl and SOCS3 reached the lowest level at day 21 and then increased gradually. The microbiome analyses revealed that the abundances of *Alistipes, Blautia*, and *Lachnospiraceae*_NK4A136_group were significantly changed at day 14, whereas the abundances of *Allobaculum, Eubacterium* and *Helicobacter* were significantly changed at day 30 of EAE induction. The prevotellaceae_NK3B31_group may be key bacteria that contribute to the development of MS. Regulation of intestinal microbiota composition can become a new therapeutic target for the treatment of MS.

## Introduction

Multiple sclerosis (MS) is a devastating autoimmune disease leading to progressive deterioration of the central nervous system (CNS) [1]. The common symptoms of MS include blindness, paresis, and sensory disturbances, accompanied by many cognitive problems such as memory disturbances and inattention [2]. The incidence of MS in women is higher than that in men and it is the most important nervous system disease causing nervous dysfunction in young adults [3,4]. Although great efforts have been mode to elucidate the underlying mechanisms, the real cause of MS is still unknown. The pathogenesis of MS has been reported to be related to many genetic and environmental factors, such as virus infection, dietary salt intake, smoking, adolescent obesity, alcoholism, and vitamin D deficiency [5–7].

The mammalian intestine is colonized by a complex microbial community, which consists of numerous microorganisms such as viruses, fungi, and bacteria [8]. Bacteria are the most abundant organisms and play essential roles in modulating host physiology such as metabolizing dietary nutrients and defending against pathogen colonization [9]. Moreover, diverse bacteria in the intestine also provide rich sources of microbial-derived antigen that can drive and promote the development of host immune system [10]. Recently, accumulating evidences have showed that the gut microbiota is another important environmental factor that is associated with the development of MS [11–13]. For instance, relapsing-remitting mice can spontaneously develop experimental autoimmune encephalomyelitis (EAE), a murine disease model that recapitulates many aspects and features of MS [13,14]. Interestingly, the EAE incidence can only occur under specific pathogen-free conditions but not germ-free conditions [13]. Furthermore, mono-colonization with segmented filamentous bacteria into the germ-free mice is sufficient to induce the development of EAE, suggesting that the activation of immune system by microbial stimulation may be a key environmental component contributing to the onset of this disease [15].

Recently, human studies also provided evidences about the associations between altered gut microbiota and the pathogenesis of MS. For example, a small sample sizes (20 MS patients versus 40 healthy subjects) study has reported that decreased abundances of *Faecalibacterium, Prevotella* and *Anaerostipes*, and increased abundances of *Bifidobacterium* and *Streptococcus* were found in MS patients compared with healthy individuals [16]. In another study with a larger sample size (60 MS patients versus 43 healthy subjects), the relative abundance of *Methanobrevibacter* and *Akkermansia* was increased, while the relative abundance of *Butyricimonas* was decreased in MS patients compared with the healthy controls [12]. A recent study also found that the relative abundances of *Akkermansia muciniphila* and *Acinetobacter calcoaceticus* were enriched in MS patients. By exposing the peripheral blood mononuclear cells to the cultures of these bacteria, the authors observed that these bacteria could stimulate the differentiation into proinflammatory Th1 lymphocytes [17].

As mentioned above, EAE is an ideal animal model that can well simulate the occurrence, development and outcome of human MS [13,15]. EAE can be induced by immunization with antigens of CNS such as myelin oligodendrocyte glycoprotein (MOG) [18]. In the present study, we used MOG-induced EAE model to assess the dynamic relationships between the composition of gut microbiota and inflammatory factor levels in lymphocytes during the onset and development of MS. We hope these findings could provide new insights into the role of intestinal flora in the pathogenesis of MS and related immunotherapy for clinical practices.

## Materials and methods

### Experimental animals

Forty-eight female C57Bl/6J mice (6–8 weeks of age) were purchased from Animal research center of Gansu University of Traditional Chinese Medicine and maintained under standard specific-pathogen free facility. Mice were randomly divided into control group by using the method of random number table.

### Establishment of EAE model

EAE was induced in mice as described previously [19]. Briefly, MOG35-55 (Synthetic Biomolecules) were diluted by 0.9% of normal saline into 10 mg/ml and then added with same volume of complete Freund’s adjuvant (CFA, Chondrex) and the same amount of tuberculin H37Ra (DIFCO, U.S.A.) to make the final concentration of tuberculin H37Ra as 4 mg/ml. Preparation of antigen emulsifier inducing EAE was made by pumping the reagent into a water-in-oil status. Mice were subcutaneously injected with 0.1 ml of emulsifier at 4 points on both sides of the spine, and then intraperitoneally injected with 0.5 ml of pertussis toxin (LBL, U.S.A.) as an immunopotentiator on the day of immunization and 48 h later, respectively.

The disease scores of neurobehavioral defects were measured with the following criteria: 0, no obvious symptoms; 1: tail paralysis; 2: hind legs weakness; 3: hindlimb paralysis; 4: limbs paralysis; 5: death.

### Extraction of total genomic DNA from feces

Mice feces were collected from control group and EAE group on day 7, 14, 21, and 30 after immunization. Total DNA of fecal samples was extracted using a QIAamp-DNA stool mini kit (Qiagen, Germany) according to the manufacturer’s instructions. Electrophoresis in 1% agarose gels was used to examine the integrity of the DNA samples, which were then selected to perform the consequent PCR amplification by using the specific primers of V4 hyper-variable regions of 16S rRNA genes. Sequencing of the PCR amplification products was performed on an Illumina Miseq platform. High quality sequencing data were analyzed and were further classified into operational taxonomic units (OTUs) with a 97% similarity level. The comparison database was Silva_12816SrRNA database. Significant difference of bacterial composition in EAE group at different time points was analyzed by Metastats software. LEfSE online analysis software was used for comparisons among different time points.

### ELISA

Cytokine (IL-17 and IFN-γ) levels in the serum of the control group and EAE group on day 7, 14, 21, and 30 after immunization were determined by ELISA kit (Neobio) according to the manufacturer’s instructions.

### Real-time PCR

Total RNA of brain paraventricular tissues were extracted from the control group and EAE group on day 7, 14, 21, and 30 after immunization. Briefly, 70 mg brain tissues were added with 1 ml Trizol reagent to extract total RNA. DNA was synthesized and amplified by PCR on LightCycler (Roche). About 20 μl of the reaction system include: 10 μl of 2x All-on-One qPCR Mix, 2 μl of DNA template, 2 μl of All-in-one qPCR Primer (Fulengen), sterilized and distilled water supplemented to 20 μl.

### Data analysis

Statistical analysis was performed through SPSS22.0. The difference between two groups was examined using un-paired Student’s *t*-test. Correlation analysis was conducted by Spearman correlation analysis. All the differences were considered to be significant at *P* < 0.05.

## Results

### Clinical symptom and body weight change

**Figure 1:**
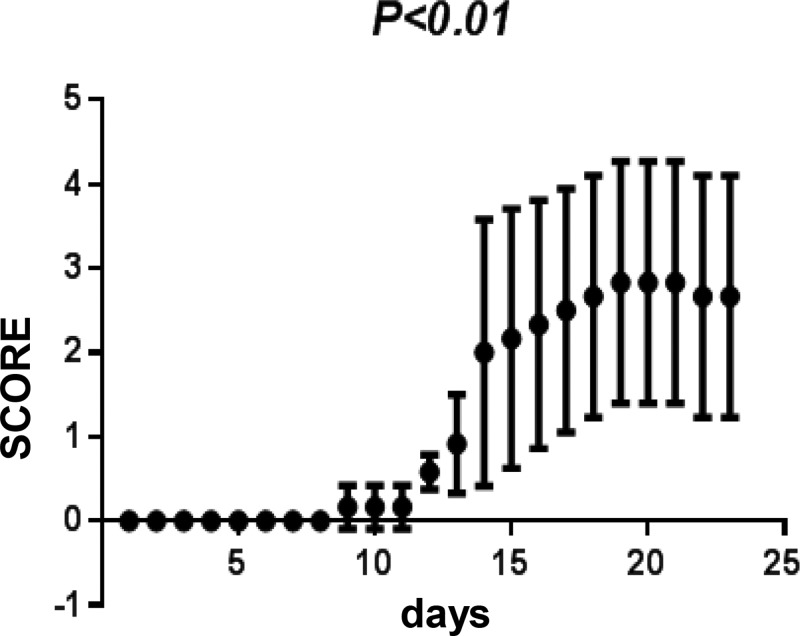
Changes of neurological function scores in control group and experimental autoimmune encephalomyelitis (EAE) group

The average onset time of EAE was on day 7 and reached the most serious level on day 21. EAE animals developed a chronic disease with the loss of appetite and body weight, as well as other symptom such as rough fur. After EAE induction, animals also displayed neurological deficits such as tail weakness, limbs paralysis, and even death. In EAE group, the neurological function scores were 2 ± 1.58 on day 14 and 2.93 ± 1.43 on day 21. There were significant differences on neurological function scores and the body weight between control group and EAE group (Table 1 and Figure 1).

**Table 1:** The body weight between control group and EAE group ($\bar{X}$S)

Group	Number	Day 14	Day 21	Day 30
EAE	24	16.28 ± 0.23	15.96 ± 0.45	18.28 ± 0.41
Normal	24	19.40 ± 0.46	20.86 ± 0.72	22.24 ± 0.50

### Serum cytokine analysis

**Figure 2:**
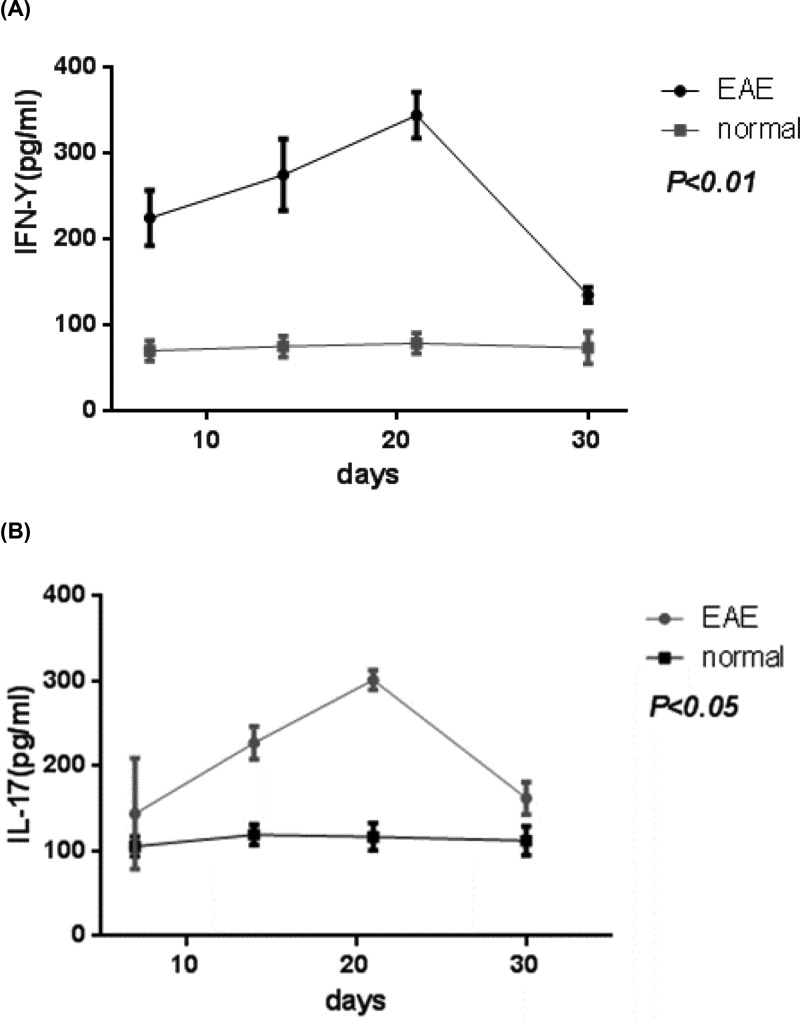
Serum cytokine analysis Serum levels of IL-17 (**A**) and IFN-γ (**B**) in control group and experimental autoimmune encephalomyelitis (EAE) group. *P* represents the comparison of day 12 and 30 with day 21 in EAE group.

In EAE group, the serum levels of IL-17 on day21 were significantly higher than that on day 14 and 30. Similar trend was also observed for the concentration of and IFN-γ (Figure 2).

### qPCR analysis

**Figure 3:**
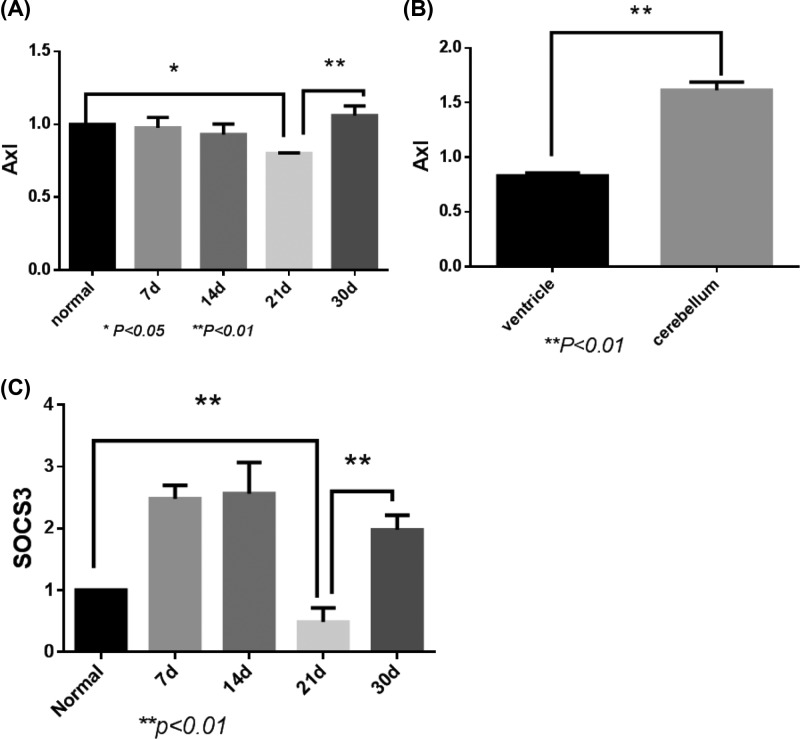
Axl and SOCS3 expressions in ventricle of the control group and experimental autoimmune encephalomyelitis (EAE) group (**A**) The expression of Axl in brain paraventricular tissues on different days of EAE induction. (**B**) Axl expression in the paraventricular and cerebellum on day 21 of EAE induction. (**C**) The expression of SOCS3 on different days of EAE induction.

In EAE group, the expression of Axl in brain paraventricular tissues started to decrease on day 7 and reached the lowest level on day 21 (Figure 3A). On day 21 of EAE induction, Axl expression was significantly lower in the paraventricular compared with that in cerebellum (Figure 3B). However, the expression of SOCS3 increased on day 7 and 14 of EAE induction, and markedly decreased on day 21 (Figure 3C). The expressions of both Axl and SOCS3 were significant different between the control group and the EAE group (Figure 3).

### Gut microbiota composition analysis

**Figure 4:**
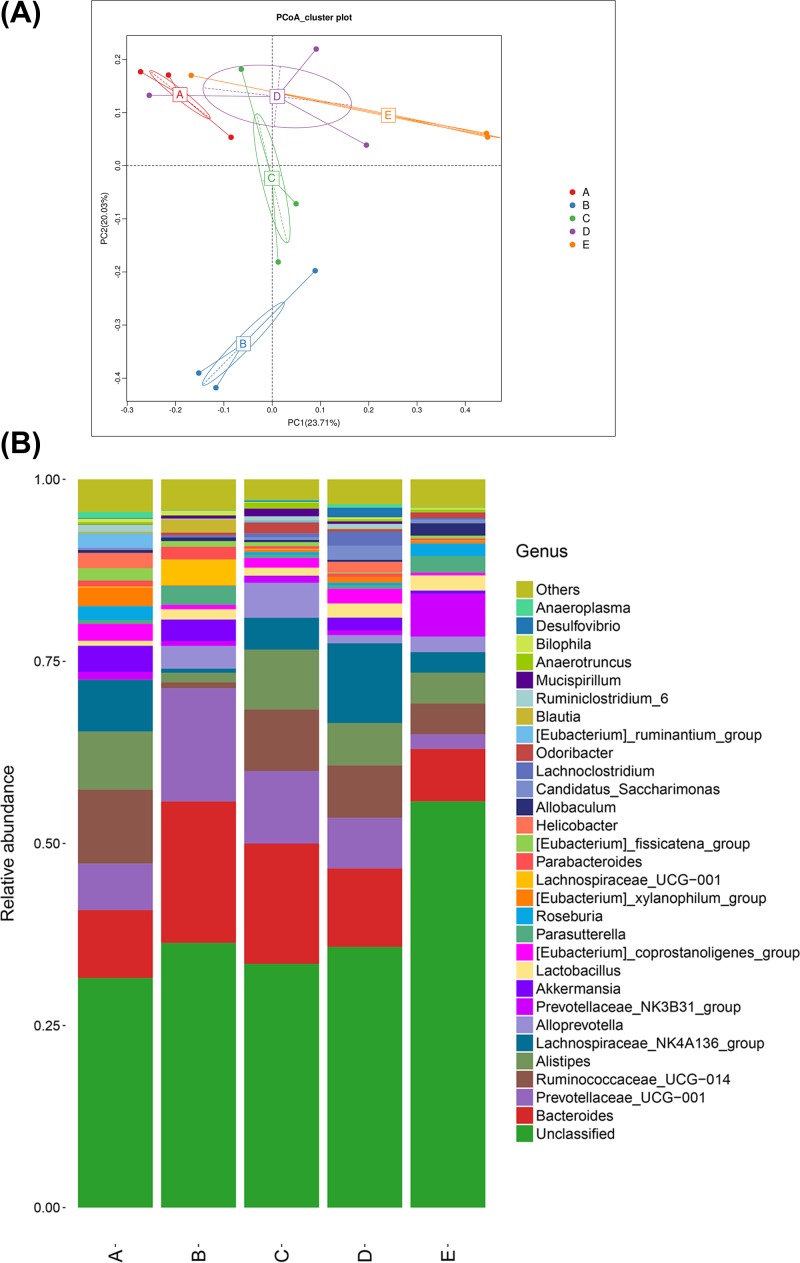
Analysis of principal component and bacterial composition differences (**A**) Principal component analysis (PCA) of the bacterial composition between the control group and experimental autoimmune encephalomyelitis (EAE) group. (**B**) The differences of bacterial composition at genus level. A–D represent day 7, 14, 21 and 30 after EAE induction in EAE group, respectively. E represents the control group.

**Figure 5:**
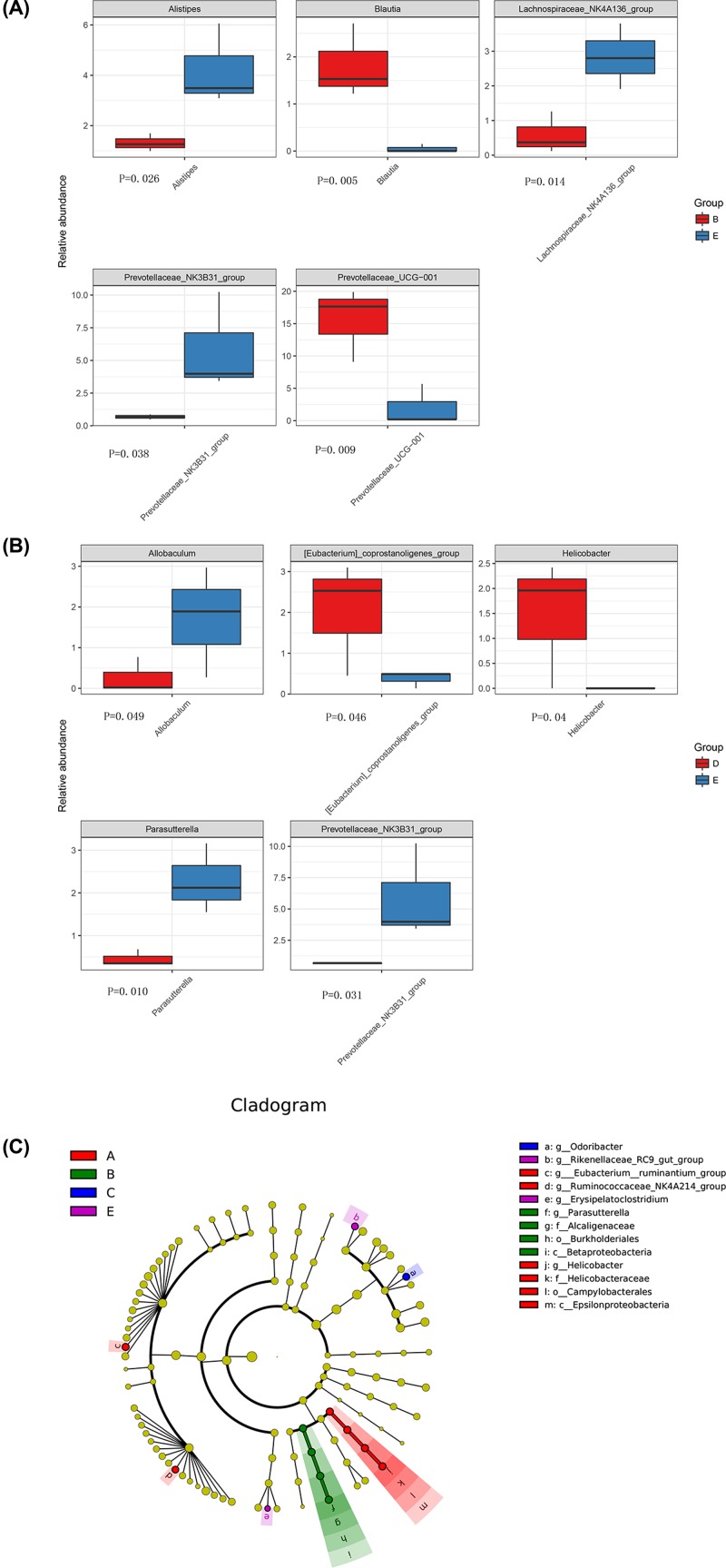
The relative abundances and LEfSE analysis (**A**) The relative abundances of *Alistipes, Blauti, Lachnospiraceae*_NK4A136_group, *prevotellaceae*_NK3B31_group, and *prevotellaceae*_UCG-001 between the control group (blue-E) and the day 14 of EAE group (red-B). (**B**) The relative abundances of *Allobaculum, Eubacterium, Helicobacter, Parasutterella*, and *Prevotellaceae* between the control group (blue-E) and the day 30 of EAE group (red-D). (**C**) LEfSE analysis of the bacterial composition on day 7, 14, 21 and 30 of the EAE group. A–D represent day 7, 14, 21 and 30 after EAE induction in EAE group, respectively. E represents the control group.

16S rDNA sequencing showed that a total of 221 OTUs with 97% similarity level were obtained from the control group and the EAE group. These OTUs included 15 kingdom, 125 phylum, 189 class, 189 order, 336 family, 688 genus, and 38 species. Principal component analysis (PCA) revealed that although individual difference existed in the EAE samples at the different time points, the bacterial composition between the EAE group and control group was still obvious (Figure 4A). At the genus level, *Bacteroides, Prevotellaceae, Ruminococcaceae, Alistipes, Lachnospiraceae*, and *Alloprevotella* were the most abundant bacteria in all samples (Figure 4B). Moreover, *Alistipes, Blauti, Lachnospiraceae*_NK4A136_group, *prevotellaceae*_NK3B31_group, and *prevotellaceae*_UCG-001 were the significantly changed bacteria between the control group and the day 14 of EAE group (Figure 5A). *Allobaculum, Eubacterium, Helicobacter, Parasutterella*, and *Prevotellaceae* were the significantly changed bacteria between the control group and the day 30 of EAE group (Figure 5B). LEfSE analysis further confirmed that *Ruminococcaceae, Epsilonproteobacteria, Campylobacterales, Helicobacteraceae*, and *Helicobacter* were the featured bacteria on day 7 of the EAE group. *Burkholderiales, parasutterel, Betaproteobacteria*, and *Alcaligenaceae* were the featured bacteria on day 14 of the EAE group. *Odoribacter* was the featured bacteria on day 30 of the EAE group (Figure 5C). It should be noted that the abundance of prevotellaceae_NK3B31_group, which was negatively correlated with serum IFN-γ level and positively correlated with Axl mRNA expression, was significantly reduced in the EAE group compared with that of the control group, suggesting that this bacteria may play an important role in the development and onset of EAE pathogenesis.

## Discussion

As the largest microbial colonization site in the human body, intestinal microbiota plays an important role in protecting intestinal barrier, inhibiting pathogenic colonization and promoting the formation of mucosa and systemic immune response [20]. There is growing evidence about the existence of Microbiome-Gut-Brin Axis and the role of gut microbiota in the pathogenesis of MS. It has been found that the abundances of *Brevibacterium methanogens* and *Ekmania*, which can activate the innate and adaptive immune system by stimulating the differentiation of T cells and monocytes, were significantly increased in the intestinal tract of MS patients [12]. However, disease-modifying treatment can inhibit the increase of prevotellaceae in MS patients [12]. These findings suggest that the increased pathogen and decreased beneficial bacteria can cause inflammatory status in the intestine and CNS, thereby leading to or aggravating the pathological changes of MS [21]. Thus, gut microbiota dysbiosis may play an important role in the course of multiple sclerosis.

It is believed that intestinal microbiota may be involved in the development of MS by regulating lymphocyte differentiation, metabolites and inflammatory factor production, as well as blood–brain barrier integrity. In the present study, we used MOG-induced EAE model to study the mechanism of how gut microbiota modulates inflammatory responses of MS onset. MOG35-55 specifically activates T lymphocytes, which may enter the CNS through damaged blood–brain barrier, causing brain lesions by releasing cytokines [22]. The mice under the induction of EAE showed chronic monophasic course and multiple different degrees of CNS lesions mainly located in white matter, cerebellum, brainstem, and spinal cord. Compared with the normal control group, HE staining of brain and spinal cord sections in EAE group at day 21 showed obvious inflammatory cell infiltration and the degree of inflammation is severed in the brain than the spinal cord. Moreover, vasodilation and ‘sleeve’ like infiltration of inflammatory cells can be seen in brain tissues, especially in subcortical and paraventricular areas. In the EAE group, the trend of body weight loss during the induction was consistent with the clinical scores.

Tyro3, Axl, and ER receptor tyrosine kinases (TAM receptors) belong to the receptor tyrosine kinase subfamily that can regulate innate immunity and mediate apoptotic cell clearance. The study found that Tyro3^−/−^, Axl^−/-^, and Mer^−/−^ mice were suffered from chronic inflammation and autoimmune diseases [23]. However, the mechanism of action is not clear. TAM receptor can suppress innate immunity by down-regulating the JAK/STAT1 signaling pathway mediated by proinflammatory cytokines IFN-α [24]. Previous studies have found that the Axl protein can bind to INF-α and activate anti-inflammatory factors SOCS1 and SOCS3 [25]. The present study found that during the EAE induction the expression of Axl was lower on day 21 than that on day 7 and 14. Consistently, the expression of anti-inflammatory factor SOCS3 increased on day 7 and 14, but decreased to the lowest level on day 21 (*P* < 0.01). However, the expression of inflammatory factors IL-17 and IFN-γ reached the highest level on day 21. These results suggest that the inflammatory demyelination down-regulates the expression of Axl, which may result from the binding to INF-α or IFN-γ receptors, thereby mediating downstream JAK/STAT1 signaling pathway. As Axl expression decreased to the lowest level on day 21, when the SOCS3 expression also reached the lowest level, which eventually led to the peak of inflammatory demyelination after in 21 days of EAE induction. Therefore, it is suggested that IFN and IL-I7 are also important factors for Axl to inhibit immune responses through JAK/STAT1-SOCS3 signaling pathway [26]. Moreover, the expression of Axl in paraventricular tissues was significantly lower than that in cerebellum, indicating that inflammatory demyelination in MS was mostly found around lateral ventricles, where the low expression level of Axl diminished the inhibitory effect on inflammatory response.

The most abundant bacteria *Bacteroides, Prevotellaceae, Ruminococcaceae, Alistipes, Lachnospiraceae*, and *Alloprevotella* observed in EAE mice are in agreement with previous study about gut microbitoa composition in MS patients [12]. Compared with the normal control group, the bacterial composition in the EAE group were significantly different at different time points, suggesting that the disturbance of gut microbiota in EAE mice is closely related to the occurrence of inflammatory demyelination. *Lachnospiraceae* belongs to the *Lachnospira* [27]. In an experimental study of MS, it was found that after giving a high-vegetable or low-protein diet in patient with RRMS, the abundance of *Lachnospiraceae* in patients increased, and the production of IL-17 by CD4^+^ T cells significantly decreased (*P* = 0.04) [28], suggesting that *Lachnospiraceae* can reduce the inflammatory response. In the present study, in the EAE group over 7 days, the abundance of *Lachnospiraceae*_NK4A136_group was found to increase and the level of IL-17 increased that can activate CD4^+^ T cells and then promote inflammatory responses. The abundance of *Helicobacter* and *Eubacterium* in the EAE group increased compared with that in the normal control group at 30 days, suggesting that they may be opportunistic pathogens in the course of EAE. Both *Blautia* and *Allobaculum* can produce short-chain fatty acids [29]. The abundance of *Blautia* increased at 7 days, but decreased thereafter, and with no significant difference at 30 days compared with the normal control group. In EAE group, the abundance of *Allobaculum* at 30 days was significantly lower than that of the normal control group. One interesting finding of the present study is that the abundance of prevotellaceae_NK3B31_group was decreased on day 14 and 30 of the EAE group compared with the control group. Prevotellaceae is believed to be associated with the synthesis of short chain fatty acids (SCFAs) [30]. Haghikia et al. [31] found that SCFAs can induce the differentiation of Treg cells, indicating SCFAs play an important role in inhibiting the immune response in CNS diseases. IFN-γ is a typical Thl-type cytokine, which promotes the release of lymphotoxin or tumor necrosis factor and causes demyelination changes in MS patients [32,33]. IL-17 is an early inflammatory cell factor with a wide range of biological activities that is secreted by Th17 cells [15]. After the intestinal microbiota is disrupted, increased IL-17 in the blood system can directly cross through the blood–brain barrier by binding with the receptor on the endothelial cells on the barrier [34]. This suggests that gut microbiota and related inflammatory mediators may participate in the development of MS by directly crossing the blood–brain barrier, changing the integrity and permeability of the blood–brain barrier, or directly stimulating various neuroimmune substances [35]. Correlation analysis showed that prevotellaceae_NK3B31_group was negatively correlated with serum IFN-γ level and positively correlated with Axl mRNA expression, indicating the existence of microbiome–gut–brin axis [36]. Moreover, the LEfSE analysis showed that *Odoribacter* was the featured bacteria that was enriched on 21 of EAE induction. However, these were only few studies about *Odoribacter* and the specific mechanism of *Odoribacter* in MS development needs to be further studied.

In conclusion, the present study that focused on the role of Axl revealed the relationship between the gut microbiota and dynamic changes of important inflammatory factors for the immune-associated development of MS. The disorder of intestinal microbiota may play an important role in the differentiation of lymphocytes and the expression of inflammatory factors in EAE mice. The roles of specific intestinal bacteria such as *prevotellaceae*_NK3B31_group and *Odoribacter* in the pathogenesis of EAE need to be further studied to explore new ways of immunotherapy.

## Abbreviations

CNS: central nervous system
EAE: encephalomyelitis
MS: multiple sclerosis
